# Outcome of Supraclavicular Artery Island Flap for Reconstruction of Head and Neck Defects in Indian Population and Their Correlation with Flap Dimensions

**DOI:** 10.1055/s-0045-1809034

**Published:** 2025-05-08

**Authors:** Sachin Verma, Sonia Moses, Saurabh Gupta, Ankit Baghel, Fareed Khan, Avinash Gautam, Ramendra Singh Gurjar

**Affiliations:** 1 Department of General Surgery, Mahatma Gandhi Memorial Medical College, Indore, Madhya Pradesh, India; 2 Department of Plastic Surgery, Mahatma Gandhi Memorial Medical College, Indore, Madhya Pradesh, India

**Keywords:** supraclavicular artery island flap, head and neck defects, flap dimensions, complications

## Abstract

**Introduction:**

This is a study to assess versatility of the supraclavicular artery island flap (SCAIF) for reconstruction of head and neck defects. This study has also evaluated complications of SCAIF and their correlation with flap dimensions.

**Materials and Methods:**

This is a prospective study done at a single institute for 1-year duration. A total of 30 patients with head and neck cancer were included in the study. SCAIF was used in all patients for reconstruction of defect after carcinoma resection. Postoperative follow-up was done for 3 weeks. The study variables were location of defect, length and width of flap, time taken for flap harvest, and complications.

**Results:**

Flap-related early complications were observed in 6 (20%) patients. These early complications were distal margin necrosis in four patients, partial flap loss in one patient, and wound infection and development of orocutaneous fistula in one patient. Stiffness in neck and shoulder movements (late complication) was noted in two patients. Among patients who developed complications, the mean flap length was 16.38 cm when compared with 15.68 cm among those who did not develop complications. Difference in mean flap length was found to be statistically significant ( *p* -value = 0.019).

**Conclusion:**

SCAIF provides a skin paddle which is hairless, thin, pliable, and color-matched with the recipient site in the head and neck region. However, its limited reach and smaller volume should be kept in mind while planning reconstruction. The safe length of SCAIF is 16 to 17 cm (up to lower third of deltoid muscle), which makes it a suitable choice for defect of lower third of the head and neck region.

## Introduction

Head and neck cancer patients after resection and neck dissection require reconstruction of the defect using local, regional, or free flap. The supraclavicular artery island flap (SCAIF) provides a versatile and reliable regional flap option for soft tissue defects in this region. SCAIF can be used to cover defects of buccal mucosa, tongue, floor of mouth, cheek, parotid, and post-auricular area. It can also be used as a reconstruction option in cases where patients have undergone multiple neck dissections or require a salvage procedure due to free flap failure.

This study was undertaken to assess the versatility of SCAIF by measuring the surgical dimensions of flap, and to evaluate the ease of flap harvest by recording time taken to harvest. Flap-related complications and their correlation with flap dimensions were evaluated. This study also provides evidence that SCAIF could be used as a first-line flap for head and neck reconstruction in selected cases.

## Materials and Methods

This prospective study was conducted in our institute for 1 year. A total of 30 patients were included in the study. Inclusion criteria for the study were patients with head and neck cancer who were scheduled to undergo wide local excision with or without neck dissection and required a flap for defect reconstruction. This applied to patients of all ages, both genders, and those who were willing to participate in the study. Patients with comorbidity like diabetes mellitus, hypertension, or poor nutrition were also included in study. Defect size varied from 5 cm × 8 cm to 7 cm × 8 cm. All defects were low volume, soft tissue only defects, without mandible resection. Those patients were excluded from the study for whom SCAIF was not found suitable to cover the expected defect while doing planning in reverse. Thus, patients with large volume defects or defects higher than lower third of face were excluded. Patients not willing for a scar at the shoulder were also excluded.

We obtained informed consent from all patients in written form, and our research and ethics committees have approved this study. Reconstruction was done with supraclavicular island flap in all patients. Flap surgery was performed by a team of two plastic surgeons in all patients. During early postoperative period, neck was supported with a sandbag in opposite side to make sure that the neck was kept in neutral position or flexed to the side of flap harvest. Patients without any flap-related complications were discharged after first dressing on postoperative day 5. Examination on follow-up visits was done on 7th, 14 ^th^ , and 21st day to assess for any complications. The study variables (location of defect, length and width of flap, time taken for flap harvest, and complications) were noted in the working proforma. Flap size and harvest time in patients who developed complications were compared with those who did not develop complications using independent *t* -test. *p* -Value less than 0.05 was considered as statistically significant.

### Surgical Technique

The patient was lying in supine position with a sandbag placed under ipsilateral shoulder and neck turned to opposite side. After cancer resection the defect was measured and the flap was planned in reverse. The posterior triangle of neck (clavicle, sternocleidomastoid, and trapezius) was marked. The external jugular vein was marked crossing the posterior triangle. The supraclavicular vessel was marked based on anatomical landmarks. The vessel is located behind the belly of omohyoid muscle in the marked triangle. The vessel runs toward the acromion process, parallel to the clavicle. The upper extent of the flap was marked by superior margin of the trapezius muscle. The lower extent was marked as per required flap width, which could be maximum 8 cm. The lateral extent of flap was marked over the deltoid muscle, as per required pedicle length, to reach the defect.

**Fig. 1:**
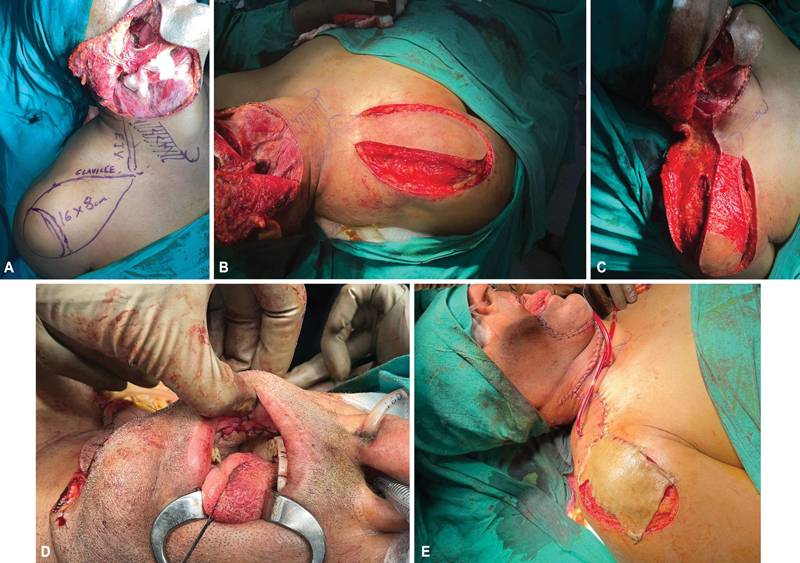
**(A)** Right buccal mucosa defect after resection of carcinoma and neck dissection with markings of supraclavicular artery island (SCAI) flap. **(B)** SCAI flap elevated. **(C)** Pedicle area de-epithelialized. **(D)** Flap inset completed. **(E)** Distal part of flap donor site covered with split skin graft.

After marking the flap was harvested in lateral to medial direction including the fascia over deltoid muscle. Once the clavicle is reached, then the periosteum was incised and included in the flap. Further dissection was done carefully avoiding injury to the flap vessel. No attempt was made to skeletonize the vessel. A width of around 5 cm was maintained for the pedicle while dissecting. Skin over the pedicle was de-epithelialized. The flap was transferred to the defect through a tunnel. The tunnel was created over the mandible in subcutaneous space for buccal mucosa defects. However, the tunnel was created under the mandible in the floor of mouth for defects of tongue. The harvested flap was then placed into the defect and inset given. A suction drain was placed at the donor area. The donor wound was closed primarily in proximal part and covered with a split-thickness skin graft distally ( Figs. 1 2 3 ).

**Fig. 2:**
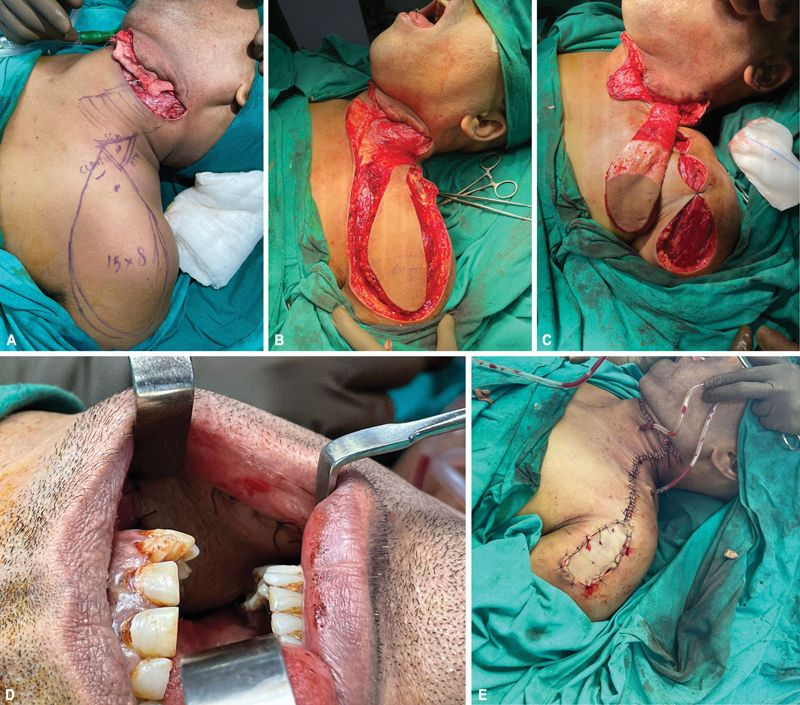
**(A)** Left buccal mucosa defect after intraoral resection of carcinoma and neck dissection with markings of supraclavicular artery island (SCAI) flap. **(B)** SCAI flap elevated. **(C)** Pedicle area de-epithelialized. **(D)** Flap inset completed. **(E)** Distal part of flap donor site covered with split skin graft.

**Fig. 3:**
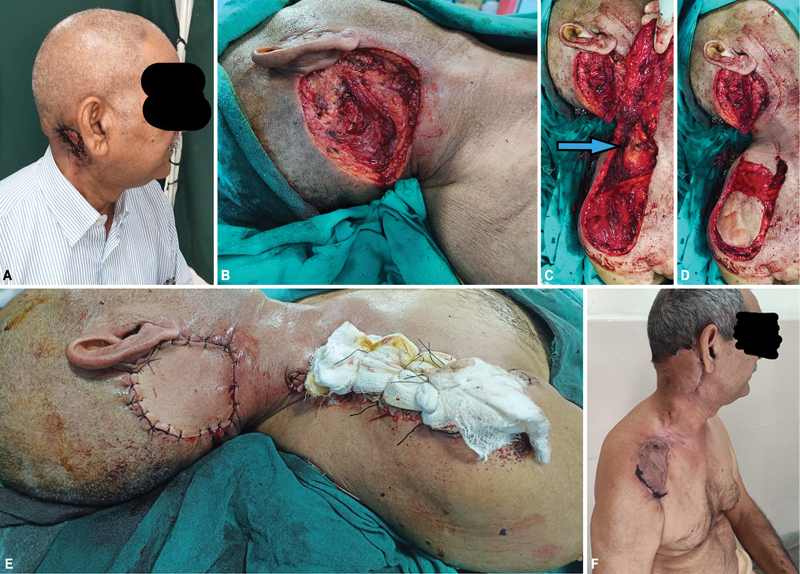
**(A)** Carcinoma in postauricular area. **(B)** Postauricular defect after resection of carcinoma. **(C)** Supraclavicular artery island (SCAI) flap elevated. Arrow showing vessel covered with fat pad on undersurface of flap. **(D)** Pedicle area de-epithelialized. **(E)** Flap inset completed and distal part of donor site covered with split skin graft with tie over dressing. **(F)** Three-week follow-up showing healthy flap and donor site. Patient is able to extend his neck comfortably.

## Results

A total of 30 patients were included in our study, out of which 21 patients were males and 9 were females. Age ranged from 28 to 71 years. Most patients were in the age group 45 to 60 years (63%, *n* = 19). Location of the lesion was buccal mucosa in 21 (70%) patients, tongue and floor of mouth in 8 (26.7%) patients, and post-auricular area in 1 (3.3%) patient ( Table 1 ).

**Table 1:** Master chart of observations

S. No.	Age (y)	Gender	Location of defect	Width of flap (cm)	Flap skin paddle length (cm)	Flap pedicle length (cm)	Total length of flap (cm)	Total flap area (sq. cm)	Time taken in flap harvest (min)	Complication
1	71	F	Tongue	8	5	11	16	128	40	Absent
2	45	M	Buccal mucosa	8	5	11	16	128	38	Absent
3	48	M	Buccal mucosa	8	6	11	17	136	40	Absent
4	45	M	Buccal mucosa	8	5	10	15	120	36	Absent
5	56	M	Buccal mucosa	8	6	11	17	136	42	Partial flap necrosis
6	64	F	Buccal mucosa	8	6	11	17	136	44	Neck stiffness
7	58	M	Tongue	8	6	11	17	136	40	Absent
8	54	M	Buccal mucosa	8	6	11	17	136	40	Infection and wound dehiscence and orocutaneous fistula
9	56	M	Buccal mucosa	8	6	11	17	136	38	Distal marginal necrosis
10	53	M	Buccal mucosa	8	5	11	16	128	38	Absent
11	54	M	Post-auricular	8	5	11	16	128	40	Absent
12	28	F	Buccal mucosa	8	5	10	15	120	36	Absent
13	56	F	Buccal mucosa	8	4	11	15	120	36	Absent
14	62	M	Buccal mucosa	8	5	11	16	128	36	Distal margin necrosis
15	58	M	Tongue	8	5	10	15	120	36	Absent
16	62	F	Buccal mucosa	8	5	10	15	120	36	Distal margin necrosis
17	64	F	Tongue	8	5	10	15	120	38	Absent
18	66	M	Buccal mucosa	8	5	10	15	120	36	Absent
19	55	M	Tongue	8	5	10	15	120	36	Absent
20	60	F	Tongue	8	5	11	16	128	38	Neck stiffness
21	48	M	Buccal mucosa	8	5	10	15	120	36	Absent
22	46	F	Buccal mucosa	8	6	11	17	136	38	Absent
23	52	M	Buccal mucosa	8	5	10	15	120	38	Absent
24	60	M	Tongue	8	5	11	16	128	40	Distal margin necrosis
25	56	M	Buccal mucosa	8	5	11	16	128	38	Absent
26	60	M	Buccal mucosa	8	6	11	17	136	38	Absent
27	58	M	Tongue	8	6	10	16	128	40	Absent
28	68	M	Buccal mucosa	8	5	10	15	120	40	Absent
29	58	F	Buccal mucosa	8	6	10	16	128	38	Absent
30	64	M	Buccal mucosa	8	5	10	15	120	36	Absent

Flap dimensions were 15 × 8 (120 sq.cm) in 12 (40.0%) patients, 16 × 8 (128 sq.cm) in 10 patients (33.3%), and 17 × 8 (136 sq.cm) in 8 patients. The mean flap size was 126.9 sq.cm. Mean time for harvesting flap was 38.20 minutes ( Table 1 ).

A total of 22 patients did not have any complication of surgery. However, flap-related early complications were observed in 6 (20%) patients. Distal margin necrosis was observed in four patients and partial (distal third) flap loss was observed in one patient. These five flaps were managed with local wound care and healed by secondary intention. In one patient, there was wound infection, pus discharge, and development of orocutaneous fistula. This fistula was closed using tongue flap. Stiffness in neck and shoulder movements (late complication) was noted in two patients, which was relieved with physical therapy.

Among patients who developed complications, the mean size was 131.0 sq.cm when compared with 125.4 sq.cm among those who did not develop complications. The difference between the mean flap sizes in both groups was found to be statistically significant using an independent sample *t* -test ( *p* -value = 0.04; Table 2 ).

**Table 2:** Comparison of mean size of flap with development of complications

Complications	* N*	Mean size of flap	SD	* t* -Statistic	* p-* Value
No	22	125.45	6.23	2.226	0.04
Yes	8	131.00	5.95		

Abbreviation: SD, standard deviation.

Among patients who developed complications, the mean flap length was 16.38 cm when compared with 15.68 cm among those who did not develop complications. The difference between the mean flap lengths of both groups was statistically significant using the independent sample *t* -test ( *p* -value 0.019; Table 3 ).

**Table 3:** Comparison of mean length of flap with development of complications

Complications	* N*	Mean flap length	SD	* t-* Statistic	* p-* Value
No	22	15.68	0.78	2.177	0.019
Yes	8	16.38	0.74		

Abbreviation: SD, standard deviation.

Flap width was kept 8 cm in all patients. Donor site was closed primarily in its proximal part and split skin graft was applied in the distal part of donor site in all patients. No donor site complication was observed in all patients.

Among patients who developed complications, the mean time was 39.25 minutes when compared with 37.82 minutes among those who did not develop complications. The difference between the mean duration of harvesting in both groups was found to be statistically insignificant using an independent sample *t* -test ( *p* -value 0.208; Table 4 ).

**Table 4:** Comparison of mean time to harvest flap with development of complications

Complications	* N*	Mean duration of harvesting	SD	* t-* Statistic	* p-* Value
No	22	37.82	1.662	1.356	0.208
Yes	8	39.25	2.816		

Abbreviation: SD, standard deviation.

## Discussion

Lamberty named the supraclavicular flap in 1983. 1 The supraclavicular artery is a branch of transverse cervical artery that originates from the thyrocervical trunk. The supraclavicular artery generally arises 3 to 5 cm from the origin of the transverse cervical artery within the triangle formed by the clavicle, sternocleidomastoid muscle, and external jugular vein. 2 3 4 5 6 7 8 9 10 11 The supraclavicular artery's origin is located 6 to 8.5 cm away from the sternoclavicular joint. The starting location of the supraclavicular artery and the point of deep fascia penetration are separated by 2 to 4.5 cm. 4 Cadaver study has demonstrated that the normal arterial diameter ranges from 1.1 to 1.5 mm. 4

Computed tomographic angiography of fresh cadaver tissue has established that the supraclavicular artery is the flap's primary source of perfusion and that the flap's distal portion depends on inter-perforator flow from the direct connecting arteries and recurrent flow through the subdermal plexus. 12 These vascular studies also recommend flap dimensions, with a mean length of 24.2 cm (Western population) and a mean width of 8.7 cm, as a guide to the size of the flap based on perfusion. 12 The study performed by Kokot et al documented the average dimensions of the flap, which were 6.1 cm in width and 21.4 cm in length. 6

Our study shows that a flap length of 15 to 17 cm is adequate to reach most defects of buccal mucosa, tongue, parotid, and postauricular area. We have observed increased rate of complications with flap length more than 16 cm (area 128 sq.cm). In our experience, the lateral limit of flap is lower third of deltoid muscle, which is less than 17 cm from the flap axis in our patient population. The safe length can be increased by tissue expansion or flap delay as suggested by Telang et al. 13 Telang et al suggest a safe length up to 20 cm even with the use of tissue expansion. 13 Other Indian studies also suggest that the distal limit of flap is up to deltoid insertion, and the average flap length is less than 17 cm in all these studies. 14 15 16 17 18 This variation in safe flap length may be due to anthropological difference between Indian and Western population. Further studies are needed to confirm this observation.

The width of flap should be kept up to 8 cm to prevent donor-site complications. Few previous studies suggest that the donor site of SCAIF up to 10 cm can be closed primarily. 3 5 6 7 We have kept the flap width of 8 cm in all our cases and the split thickness skin graft was needed in the distal portion of donor site in all cases. None of our patient had any complication related to donor site. In our experience, primary closure is under tension at the distal portion of donor site even if the width is kept 8 cm. However, the present study does not have any observation to comment on the width of donor site to be able to achieve primary closure without complications.

Supraclavicular vessels lie in the lower part of posterior triangle (level Vc of cervical lymph nodes). This vessel is usually spared in neck dissection up to level V. Studies have shown that the supraclavicular vessel is fairly reliable despite prior radiation or neck dissection. 11 19 20 Thus, SCAIF can be safely used in cases of cancer recurrence or flap failure. However, our study does not include any such patient.

The SCAIF can be raised within 40 minutes, which is comparable to other regional flaps. It has a shorter total operative time compared with microvascular flaps. 21 22 23 Flap harvest time increases with increasing size of the flap; however, it does not have significant correlation with development of complications. Being a pedicle flap, SCAIF has advantage of less need for close monitoring and lower overall expenditure compared with a free flap. 21 22 23 Free flap is not preferred in patients with advanced age, poor nutrition, or multiple comorbidities. 5 Also, the microvascular free flaps need higher expertise and resources compared with pedicled flap.

The SCAIF should not be considered superior to other regional flaps, but rather a complementary alternative reconstruction option. Regional muscle flaps (pectoralis major, deltopectoral, trapezius, and latissimus flaps) are bulky. 5 Pectoralis major myocutaneous (PMMC) flap is suitable to fill large volume defects, while the SCAIF is suitable for limited volume defects. Muscle in the PMMC flap gets atrophied, leading to a decreased range of neck movements. 7 The range of internal rotation and adduction of the shoulder is reduced after PMMC flap harvest. 5 7 SCAIF preserves the muscle and has less impact on neck movements. Another fasciocutaneous regional flap option for defects in this region is submental flap. SCAIF provides non-hair-bearing skin and larger tissue volume compared with submental flap. 9 The deltopectoral flap has a disadvantage of limited arc of rotation because of its broad base and it cannot reliably reach oral cavity or mastoid defects; however, SCAIF can reliably reconstruct these defects. 9

Limitations of SCAIF include its limited reach up to the neck and lower third of the face and potential donor-site morbidity due to scarring. 9 Donor-site scar may be visible while wearing clothes with exposed shoulder. 13 It may cause pain or irritation due to brasserie strap in females. 23

## Conclusion

The SCAIF enables one-stage reconstruction of head and neck defects with a low learning curve. It yields low cost, less time consuming, cosmetic option with manageable less severe complications that too have low chance to occur. SCAIF provides a skin paddle which is hairless, thin, pliable, and color-matched with the recipient site in the head and neck region. However, its limited reach and smaller volume should be kept in mind while planning reconstruction. Safe length of SCAI flap is 16 to 17 cm (up to lower third of deltoid muscle), which makes it a suitable choice for defect of lower third of head and neck region. A reconstruction surgeon should keep SCAIF as an option in his armamentarium.
